# Sex-related pharmacokinetic and pharmacological responses to 4F-furanylfentanyl

**DOI:** 10.3389/fphar.2026.1745891

**Published:** 2026-03-06

**Authors:** Sabrine Bilel, Camilla Montesano, Micaela Tirri, Giorgia Corli, Marta Bassi, Nicole Cocita, Fabiana Di Rosa, Adolfo Gregori, Claudio Trapella, Manuel Sergi, Matteo Marti

**Affiliations:** 1 Department of Translational Medicine, Section of Legal Medicine and LTTA Centre and University Center of Gender Medicine, University of Ferrara, Ferrara, Italy; 2 Department of Chemistry, Sapienza University of Rome, Rome, Italy; 3 Carabinieri, Department of Scientific Investigation (RIS), Rome, Italy; 4 Department of Environmental and Prevention Sciences, University of Ferrara, Ferrara, Italy; 5 Collaborative Center for the Italian National Early Warning System (NEWS-D), Department of Anti-Drug Policies and other addictions, Presidency of the Council of Ministers, Rome, Italy

**Keywords:** fentanyl, fentanyl analogs, μ-opioid receptor, novel psychoactive substances, novel synthetic opioids, pharmacokinetics, respiratory depression, sex-specific differences

## Abstract

**Background:**

Novel synthetic opioids (NSOs), including a variety of fentanyl analogs (FAs) and emerging non-fentanyl compounds, have increasingly been implicated in overdose fatalities worldwide. Among these, 4-fluoro-furanylfentanyl (4F-FUF) is a potent FA with limited *in vivo* pharmacotoxicological characterization. In this study, we aimed to i. evaluate the pharmacotoxicological effects of 4F-FUF in male and female mice, ii. determine its pharmacokinetic profile in plasma and tissues of both sexes, and iii. correlate behavioral and physiological responses with plasma concentrations.

**Methods:**

Female and male mice were injected intraperitoneally with 4F-FUF at an effective dose of 5 mg/kg. Behavioral and physiological responses, including sensorimotor, motor, and respiratory parameters, were assessed at multiple timepoints post-administration. Plasma and tissue samples (brain, heart, liver, spleen, lung, kidney, and stomach) were collected to determine 4F-FUF concentrations and pharmacokinetic parameters. Correlations between plasma levels and behavioral or physiological outcomes were analyzed separately by sex.

**Results:**

4F-FUF impaired the sensorimotor and motor responses in females and males. Moreover, the FA induced persistent antinociception in males with respect to females. The respiratory depression was sudden and more pronounced in male mice. Plasma concentrations of 4F-FUF were higher and persisted longer in males, indicating slower clearance than in females. This drug was highly distributed in the brain and liver tissues of both sexes. Significant correlations were detected in visual placing, vibrissae responses, spontaneous locomotion, and mechanical analgesia in both sexes. Interestingly, the respiratory rate was only significantly correlated with plasma concentrations in females, highlighting potential sex-specific differences in the relationship between drug exposure and physiological effects.

**Conclusion:**

The findings demonstrate marked sex-specific differences in the behavioral and physiological changes and pharmacokinetics of 4F-FUF. These results underscore the importance of considering sex-specific differences in assessing the toxicity and risk profiles of novel synthetic opioids.

## 1 Introduction

Fentanyl analogs (FAs) are a group of potent synthetic opioids that are chemically similar to fentanyl but exhibit varying potencies and effects. While some have legitimate medical uses in human and veterinary medicine, many are manufactured illicitly and are a major contributor to the ongoing opioid overdose crisis due to their unpredictable and extreme potency (United Nations Office on Drugs and Crime-UNDOC, 2023; 2024; European Union Drugs Agency-EUDA, 2024). Since 2013, the number of FAs has increased exponentially in Europe, the United States, and many other countries (Armenian et al., 2018). Fentanyl and 13 of its derivatives are currently controlled under Schedule I of the 1961 Single Convention on Narcotic Drugs. Fentanyl precursors NPP and ANPP are not controlled across the EU member states, which may explain the continued detection of fentanyl and its analogs in many fatal cases in Europe (European Union Drugs Agency, 2025).

**FIGURE 1:**
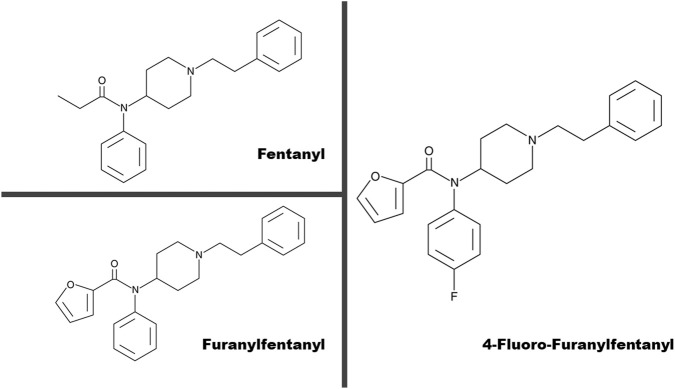
Chemical structures of fentanyl, furanylfentanyl, and 4-fluoro-furanylfentanyl.

Among the emerging FAs, 4-fluoro-furanylfentanyl (4F-FUF) was first reported in the United States in 2018 and subsequently detected in Europe in 2019 and found to be associated with serious adverse events and deaths. This FA was identified by the Italian “Carabinieri” in a 2019 Italian drug seizure packaged as “iBF 1:10 – tetra ammonium salt.” In 2022, 4F-FUF was identified in post-mortem samples of fatal cases in San Francisco (Yu et al., 2024). This opioid differs from fentanyl by a furan-2-carboxamide instead of the propionamide group and a fluorine atom in the para-position on the aromatic group (Figure 1). 4F-FUF is a close derivative of furanylfentanyl formed by the addition of a fluorine atom to the aniline ring in the para-position (Figure 1). Many studies regarding fluorinated and non-fluorinated FAs (e.g., 2-fluoro-fentanyl and fentanyl, butyrylfentanyl and fluoro-butyrylfentanyl, and valerylfentanyl and fluoro-valerylfentanyl) show that the potency of a fluorinated compound could be less, similar, or slightly higher than that of its non-fluorinated counterpart (Wilde et al., 2019; Varshneya et al., 2023, Canfield and Sprague, 2025). In addition, the isomer position (ortho, meta, or para) of the fluorine atom is crucial for modulating μ-opioid receptor-mediated effects (Varshneya et al., 2023). 4F-FUF was characterized using several analytical techniques, and the metabolic profile was elucidated *in vitro* and *in vivo* (Montesano et al., 2021; Rodriguez Salas et al., 2022; Vincenti et al., 2020).

Sex-specific differences in the pharmacotoxicological effects of opioids have been reported in scientific literature (Bilel et al., 2025a; Bilel et al., 2025b; Di Francesco et al., 2024; Fattore et al., 2020; Little and Kosten, 2023). In particular, research on sex-based treatment differences in opioid use disorders has increased; however, the findings of the studies focusing on sex-dependent differences in the pharmacotoxicological aspects of novel synthetic opioids (NSOs), particularly the class of FAs, have not yet been disclosed (Bilel et al., 2025a; Bilel et al., 2025b; Di Francesco et al., 2024; Fattore et al., 2020). Moreover, the sex-specific differences in the pharmacokinetics of the potent FAs are barely considered in recent studies. To this end, this study is aimed at i. evaluating the pharmacotoxicological effects of 4F-FUF *in vivo* in female and male mice injected intraperitoneally with an effective dose of 5 mg/kg, ii. determining the pharmacokinetics profile in plasma and tissues of both sexes, and iii. correlating the behavioral and physiological effects with the 4F-FUF levels in plasma of both sexes.

This research study is the result of collaboration between forensic research laboratories and the Scientific Investigation Unit of the Arma dei Carabinieri (RIS) in compliance with the point n°6 (preclinical evaluation of the pharmacotoxicological effects of synthetic opioids and interaction with new substances) of the “National Prevention Plan against the Misuse of Fentanyl and Other Synthetic Opioids” launched by the Department of Anti-Drug Policies and Other Addictions of the Presidency of the Council of Ministers (Italy).

## 2 Materials and methods

### 2.1 Animals

Forty female and male ICR (CD-1®) mice weighing 30–35 g (Centralized Preclinical Research Laboratory, University of Ferrara, Italy) were group-housed (five mice per cage; the floor area per animal was 80 cm^2^; the minimum enclosure height was 12 cm), exposed to a 12:12-h light–dark cycle (light period from 6:30 a.m. to 6:30 p.m.) at a temperature of 20 °C–22 °C and humidity of 45%–55%, and were provided *ad libitum* access to food (Diet 4RF25 GLP; Mucedola, Settimo Milanese, Milan, Italy) and water. The experimental protocols performed in the present study were in accordance with the UK Animals (Scientific Procedures) Act of 1986 and associated guidelines and the new European Communities Council Directive of September 2010 (2010/63/EU). Experimental protocols were approved by the Italian Ministry of Health (License No. 223/2021-PR, CBCC2.46. EXT.21) and by the Animal Welfare Body of the University of Ferrara. According to the ARRIVE guidelines, all possible efforts were made to minimize the number of animals used and pain and discomfort experienced by the animals. In alignment with our commitment to ethical research practices, we are dedicated to reducing the use of animals in our study by adhering to the principles of the 3Rs: eight male and eight female mice were used for behavioral studies (safety pharmacology), and four male and four female animals were used for the pharmacokinetic studies.

### 2.2 Drug preparation

4F-FUF was purchased from Cayman Chemical (Ann Arbor, Michigan, USA). It was dissolved in absolute ethanol (final concentration of 2% v/v) and Tween 80 (2% v/v) and diluted to its final volume with saline (0.9% NaCl v/v). The solution made with ethanol, Tween 80, and saline was also used as the vehicle (blank control). The drugs were administered by an intraperitoneal injection at a volume of 4 μL/g; the final concentration of 4F-FUF was 5 mg/kg. The control groups of female and male mice were administered only with the vehicle solution. An effective dose of 5 mg/kg was selected based on our previous studies (Bilel et al., 2022; Bilel et al., 2025a; Montesano et al., 2021) and preliminary experiments. This dose represents a sufficiently high dose to allow quantifiable measurement of 4F-FUF and its metabolites in the blood at the selected timepoints. Additionally, this dose was effective in both sexes, producing significant and sustained effects that facilitated the assessment of potential sex-specific differences in the pharmacokinetic and behavioral responses of mice.

### 2.3 Behavioral tests

In the present study, the effect induced by 4F-FUF on behavioral responses was analyzed using a battery of tests that are widely used in studies of “safety pharmacology” in rodents (Bilel et al., 2020; 2021; 2022). To reduce the number of animals used, mice were evaluated in functional observational tests carried out in a consecutive manner according to the following time scheme: observation of visual object responses (frontal and lateral view), tactile response (vibrissae reflexes), visual placing response, respiratory rate, mobility time, and tail pinch test. Behavioral tests were conducted in a thermostat-controlled (temperature: 20 °C–22 °C; humidity: 45%–55%) and light (150 lux) room with a background noise of 40 ± 4 dB. The apparatus for sensorimotor and physiological tests consisted of an experimental chamber (350 mm × 350 mm × 350 mm) with black methacrylate walls and a transparent front door. During the week before the experiment, each mouse was placed in the box and handled (once a day) every other day, i.e., three times, to acclimate to both the environment and the experimenter. To avoid mice olfactory cues, cages were carefully cleaned with dilute (5%) ethanol solution and rinsed with water. All experiments were performed between 8:30 a.m. and 2:00 p.m. and were conducted in a blinded manner by trained observers working in pairs. The behavior of the mice was videotaped using a camera (B/W USB camera day and night with varifocal lens; Ugo Basile, Italy) placed at the top or on one side of the box and analyzed offline by a different trained operator.

#### 2.3.1 Evaluation of the visual response

Visual response was verified by using two behavioral tests that evaluate the ability of the animal to capture visual information when the animal is moving (the visual placing response) or stationary (the visual object response).

The visual object response test was performed to evaluate the ability of the mouse to detect an object approaching from the front (frontal view) or from the side (lateral view), which typically induces the animal to shift or turn its head or retreat. A white horizontal bar was moved toward the mouse’s head from the front, while a small dentist’s mirror was moved laterally into the mouse’s field of view in a horizontal arc until the stimulus was positioned between the mouse’s eyes. The procedures were conducted bilaterally and repeated three times (Bilel et al., 2020). A score of 1 was assigned if movement was observed or 0 if there was no movement. The total score was calculated by adding the scores obtained in the frontal and lateral visual object response tests (overall score: 9). Tests were carried out at 10, 30, 60, 120, 180, 240, and 300 min after the injection.

The visual placing response test was performed using a modified tail suspension apparatus designed to bring the mouse down toward the floor at a constant speed of 10 cm/s (Bilel et al., 2020). The downward movement of the mouse was videotaped using a camera (B/W USB camera day and night with varifocal lens; Ugo Basile, Italy) placed at the base of the tail suspension apparatus. Movies were analyzed offline by a trained operator who was blinded to the drug treatments performed to evaluate the initiation of the reaction of the mouse when lowered. An electronic ruler was used to evaluate the perpendicular distance in millimeters between the mouse’s eyes and the floor at the exact moment the reaction begins. Untreated control mice typically perceive the floor and initiate preparations to contact at a distance of approximately 28 ± 4.3 mm. Tests were carried out at 15, 35, 70, 125, 185, 245, and 305 min after the injection.

#### 2.3.2 Evaluation of tactile responses

Tactile responses were verified through vibrissae reflexes induced by the touch of a thin hypodermic needle (Bilel et al., 2020). Vibrissae reflex was evaluated by touching vibrissae (right and left) once for each side, and a score of 1 was assigned if there was a reflex (turning of the head to the side of touch or vibrissae movement) or 0 if there was no reflex (overall score: 2).

#### 2.3.3 Evaluation of the respiratory rate

The experimental protocol for the assessment of respiratory parameters in this study includes monitoring animals when they are awake, freely moving, non-invasive, and requiring minimal handling. The mice were allowed to move freely in a cage, and the respiration patterns of the mice were videotaped using a camera (B/W USB camera day and night with varifocal lens; Ugo Basile, Italy) placed above the observation cage (Bilel et al., 2025a; Corli et al., 2022). The frame-by-frame analysis allows accurate evaluation of the mouse’s respiratory rate, which was determined by counting approximately 262 ± 7 breaths per minute (brpm). The respiratory rate was measured at 15, 40, 70, 130, 150, 190, 250, and 310 min after the injection.

#### 2.3.4 Evaluation of motor activity

The mobility time test was used to evaluate the spontaneous motor activity of mice (Corli et al., 2022). The mice were allowed to move freely in a square plastic cage (60 cm × 60 cm). The observer measures the total time spent moving by the animal (when the mouse walks or moves the front legs) during a 5-min observation period. The test was performed at 15, 35, 70, 125, 185, 245, and 305 min after the injection.

#### 2.3.5 Evaluation of pain induced by a mechanical stimulus

Acute mechanical nociception was evaluated using the tail-pinch test (Bilel et al., 2025a; Vandeputte et al., 2024). A special rigid probe connected to a digital dynamometer (ZP-50N, Imada, Japan) was used to apply progressive pressure to the mouse’s tail (in the distal portion). When the mouse flicked its tail, the pressure was immediately stopped, and the digital instrument recorded the maximum pressure (g/force) supported by the tail. A cutoff (500 g/force) was set to prevent tissue damage. The test was repeated three times, and the final value was calculated by averaging the three obtained scores.

### 2.4 Statistical analysis

In sensorimotor response experiments, data are expressed in arbitrary units (visual object response and vibrissae reflexes) and percentage of baseline (visual placing response, respiratory rate, and mobility time). The tail-pinch test is expressed as Emax% which is calculated as the percentage of the maximal possible effect {EMax% = [(test − control latency)/(cutoff time − control)] × 100}.

We used the GraphPad Prism tool “Random number calculator” (graphpad.com), setting only one repetition for randomization. Hence, for the safety pharmacology study, GraphPad assigned eight subjects to two different groups (vehicle or 4F-FUF 5 mg/kg) for each sex.

Conversely, the sample size was determined by applying the prior power analysis (Faul et al., 2007). The determination of the number of animals to be used in the study was carried out as a function of the power level of the analysis (1 − β = 0.80), the significance level (α = 0.05) to be achieved, and the magnitude of the effect observed in the considered test (effect size f) using GPower 3.1 (Cohen, 1988).

All data passed the Kolmogorov–Smirnov normality test with Dallal–Wilkinson–Lillie approximation for P-values. Statistical analysis of the effects of 4F-FUF at the dose of 5 mg/kg over time was performed using a two-way ANOVA followed by a Bonferroni test for multiple comparisons. The difference was considered statistically significant for P < 0.05. Statistical analysis was performed using Prism software (GraphPad Prism, version 9, USA).

### 2.5 Pharmacokinetic studies

#### 2.5.1 Chemicals and working solutions

4F-FUF, fentanyl-d5, and norfentanyl-d5 were purchased from Cayman Chemical (Ann Arbor, Michigan, USA), and the latter were used as internal standards (ISs). Two ISs were used because both the parent drug 4F-FUF and its metabolites were monitored; fentanyl-d5 was considered suitable for 4F-FUF, while norfentanyl-d5 was more adequate for the more polar metabolites. The IS working solution (ISWS) containing both standards at 30 ng mL^−1^ was prepared in methanol:acetonitrile (1:1, v:v) containing 0.1% formic acid. Methanol, ultrapure water, acetonitrile, formic acid, and ammonium formate were obtained from Sigma-Aldrich (Milan, Italy).

#### 2.5.2 Sample collection and pretreatment

For terminal pharmacokinetic sampling, animals were anesthetized using isoflurane delivered by inhalation (2% in oxygen for induction) until loss of righting and pedal withdrawal reflexes was confirmed.

Blood samples (total volume: 500 μL) were collected by submandibular blood collection technique into 1-mL vials containing EDTA (4 mg/mL of blood) as the preservative and anticoagulant. Then, samples were centrifuged at 9,000 rpm for 8 min to obtain plasma. After each blood withdrawal, an equal volume of saline solution was subcutaneously injected into mice to maintain volume and osmotic homeostasis. Blood samples were first collected at 30 min and then at 1, 2, 3, 4, and 5 h. Animals were then euthanized by cervical dislocation, in accordance with the institutional and national guidelines. Different tissues (brain, heart, liver, kidney, spleen, and stomach) were collected. Plasma and tissues collected from male and female mice were stored at −20 °C until subsequent use in the analysis (Bilel et al., 2025a). For blood sample preparation, plasma samples were prepared by adding 50 μL plasma (containing the analytes) + 125 μL of ISWS + 25 μL MeOH/ACN 50/50 (v/v) solution 0.1% HCOOH in each Eppendorf tube.

For tissue preparation, approximately 100 mg of each tissue was weighed in a 2-mL Precellys vial; ISWS was added to reach a solvent:tissue ratio of 5:1 (v:w). The homogenate was centrifuged at 12,000 rpm for 10 min at 4 °C, and 100 µL of the supernatant was collected and diluted to 300 µL with water before injection into the LC-HRMS system.

#### 2.5.3 LC-HRMS analysis

A Thermo Fisher Scientific UltiMate 3000 RSLC system coupled with a Thermo Fisher Scientific Q-Exactive mass spectrometer (Thermo Fisher Scientific, Bremen, Germany) was used for the analysis. Chromatographic separation was carried out using an Excel 2 C18-PFP (100 × 2.1 mm ID) column from ACE (Aberdeen, Scotland) packed with particles of 2 μm, which was maintained at 35 °C at a flow rate of 0.5 mL min^−1^.

Mobile phases consisted of 0.1% (v/v) formic acid + 10 mM ammonium formate in water (phase A) and 0.1% formic acid in acetonitrile (phase B). The gradient started with 0% phase B, and these conditions were maintained for 1 min; phase B was then increased to 25% in 2 min to 35% in the following 2 min and held for 3 min. Phase B was then ramped to 50% over 1.5 min and to 100% in 0.5 min; it was kept stable for 1 min and then equilibrated to the initial conditions, yielding a total runtime of 12.5 min. The injection volume was 6 μL.

The Q-Exactive mass spectrometer was equipped with a heated electrospray ionization source (HESI-II) operated in the positive mode; mass spectra were acquired in full scan/data-dependent in the range 50–800 m/z. The operating parameters of the ion source were set as follows: spray voltage, 3.5 kV; capillary temperature, 350 °C; heater temperature, 300 °C; S-lens RF level, 60; sheath gas flow rate, 55; and auxiliary gas flow rate, 20. Nitrogen was used for spray stabilization, for collision-induced dissociation experiments in the high-energy collision dissociation (HCD) cell, and as the damping gas in the C-trap.

The instrument was calibrated in positive and negative modes on every working day. For a full scan, the resolution was 70,000 (FWHM at m/z 200), whereas automatic gain control (AGC) and the maximum injection time were set at 1 × 105 and 100 ms, respectively. In MS/MS mode, the resolution was 35,000 (FWHM at m/z 200), and three different collision energies, namely, 10, 30, and 50, were applied. An inclusion list with the masses of the previously identified metabolites was added to the methods (Bilel et al., 2025a; Montesano et al., 2021).

## 3 Results

### 3.1 Behavioral effects of 4F-FUF

**FIGURE 2:**
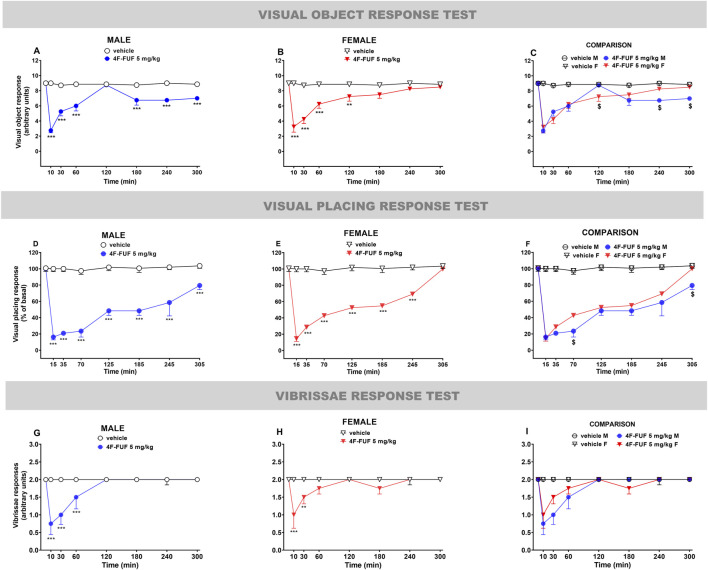
Effects of the systemic administration of 4F-FUF determined using the visual object test **(A–C)**, visual placing test **(D–F)**, and vibrissae response test **(G–I)**. Data are expressed as arbitrary units (visual object and vibrissae tests) and percentage of basal values (visual placing test) and represent the mean ± SEM of eight female and eight male mice/group. Statistical analysis was performed using two-way ANOVA followed by the Bonferroni’s test for multiple comparisons for the dose–response curve of each compound at different timepoints. **p < 0.01 and ***p < 0.001 versus vehicle. $p < 0.05 versus sex (female/male).

**FIGURE 3:**
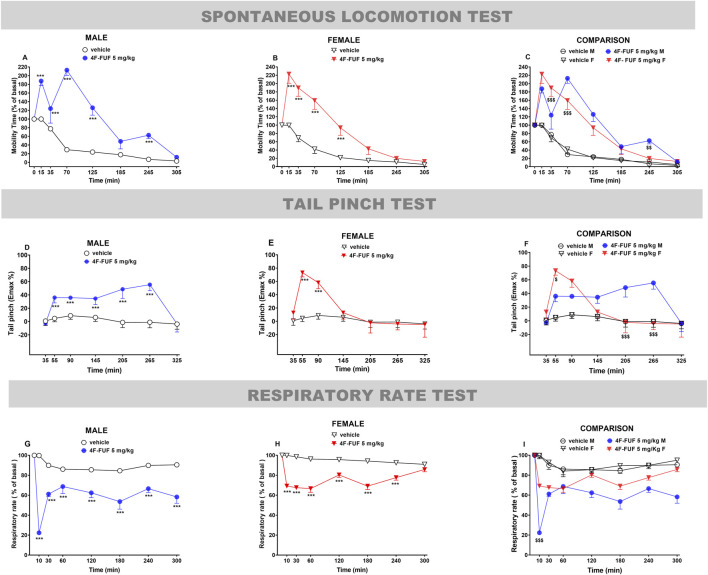
Effects of the systemic administration of 4F-FUF (5 mg/kg IP) determined using the spontaneous locomotion test **(A–C)**, tail-pinch test **(D–F)**, and respiratory rate test **(G–I)**. Data are expressed as the percentage of basal values (spontaneous locomotion and respiratory rate tests) and the percentage of maximal effects (tail pinch test) and represent the mean ± SEM of eight female and eight male mice/group. Statistical analysis was performed using two-way ANOVA followed by the Bonferroni’s test for multiple comparisons for the dose–response curve of each compound at different timepoints. ***p < 0.001 versus vehicle. $$p < 0.01 and $$$p < 0.001 versus sex (female/male).

Systemic administration of 4F-FUF (5 mg/kg IP) induced sensorimotor and motor impairments, increased analgesia, and reduced the respiratory rate in treated mice of both sexes (Figures 2, 3).

In the visual object test, the visual reflexes to the object were significantly reduced in male [Figure 2A: significant effect of treatment (F_1,112_ = 252.5; P < 0.0001), time (F_7,112_ = 22.60; P < 0.0001), and time × treatment interaction (F_7,112_ = 22.57; P < 0.0001)] and female [Figure 2B: (F_1,112_ = 158.0; P < 0.0001), time (F_7,112_ = 19.61; P < 0.0001), and time × treatment interaction (F_7,112_ = 18.81; P = 0.9998)] mice after 4F-FUF treatment. The effect was more persistent in males (up to 300 min) than in females (120 min). Sex-specific differences were determined using ANOVA at 120 min, 240 min, and 300 min of measurements [Figure 2C: F_2,168_ = 101.3; P < 0.0001)].

In the visual placing test, the visual reflexes to the floor were significantly reduced in male [Figure 2D: significant effect of treatment (F_1,112_ = 306.6; P < 0.0001), time (F_7,112_ = 13.70; P < 0.0001), and time × treatment interaction (F_7,112_ = 11.85; P < 0.0001)] and female [Figure 2E: significant effect of treatment (F_1,112_ = 752.5; P < 0.0001), time (F_7,112_ = 51.90; P < 0.0001), and time × treatment interaction (F_7,112_ = 45.31; P < 0.0001)] mice after 4F-FUF injection. In addition, in this test, the effect was more persistent in males (up to 305 min) than in females (245 min). The effect in the visual placing test was more pronounced than that observed in the visual object test in both sexes. In this case, sex-specific differences were determined using ANOVA at 70 min and 305 min of measurements [Figure 2F: F_2,168_ = 248.4; P < 0.0001].

In the vibrissae response test, the administration of 4F-FUF significantly reduced the vibrissae reflexes of male [Figure 2G: significant effect of treatment (F_1,112_ = 25.17; P < 0.0001), time (F_7,112_ = 7.10; P < 0.0001), and time × treatment interaction (F_7,112_ = 7.10; P < 0.0001)] and female [Figure 2H: significant effect of treatment (F_1,112_ = 15.74; P = 0.0001), time (F_7,112_ = 3.93; P = 0.0007), and time × treatment interaction (F_7,112_ = 3.93; P = 0.0007)] mice to touches. The effect of 4F-FUF appeared only for 60 min in both sexes and tended to reach basal values thereafter. No sex-specific differences were detected using ANOVA in this test (Figure 2I).

In the spontaneous locomotion test, the systemic administration of 4F-FUF (5 mg/kg IP) induced a significant facilitation of the motor activity of male [Figure 3A: significant effect of treatment (F_1,112_ = 124.1; P < 0.0001), time (F_7,112_ = 34.31; P < 0.0001), and time × treatment interaction (F_7,112_ = 13.32; P < 0.0001)] and female [Figure 3B: significant effect of treatment (F_1,112_ = 104.5; P < 0.0001), time (F_7,112_ = 46.21; P < 0.0001), and time × treatment interaction (F_7,112_ = 11.05; P < 0.0001)] mice. The effect was more persistent (up to 305 min) in males than in females (245 min). The effect disappeared in females after 125 min; however, in males, a significant increase was detected at 245 min. In this test, sex-specific differences were determined using ANOVA at 35, 70, and 245 min of measurements [Figure 3C: F_2,184_ = 51.79; P < 0.0001].

In the tail-pinch test, the systemic administration of 4F-FUF (5 mg/kg IP) significantly increased antinociception in male [Figure 3D: significant effect of treatment (F_1,112_ = 39.57; P < 0.0001), time (F_7,112_ = 4.84; P = 0.0002), and time × treatment interaction (F_7,112_ = 3.94; P = 0.0014)] and female [Figure 3E: significant effect of treatment (F_1,112_ = 13.19; P = 0.0004), time (F_7,112_ = 6.93; P < 0.0001), and time × treatment interaction (F_7,112_ = 4.47; P = 0.0002)] mice. The effect was more persistent in males (up to 265 min) than in females (90 min). Sex-specific differences were determined using ANOVA at 55, 205, and 265 min of measurements [Figure 3F: F_2,184_ = 14.96; P < 0.0001].

In the respiratory rate test, the systemic administration of 4F-FUF (5 mg/kg IP) significantly decreased the respiratory rate in male [Figure 3G: significant effect of treatment (F_1,112_ = 93.37; P < 0.0001), time (F_7,112_ = 6.75; P < 0.0001), and time × treatment interaction (F_7,112_ = 6.68; P = 0.0014)] and female [Figure 3H: significant effect of treatment (F_1,112_ = 123; P < 0.0001), time (F_7,112_ = 6.26; P < 0.0001), and time × treatment interaction (F_7,112_ = 6.19; P < 0.0001)] mice. The effect was more pronounced in males at 10 min than in females. A reduction in the respiratory rate persisted in males for up to 300 min, while this effect was not present in females at the same timepoint. Sex-specific differences were determined using ANOVA at 10 min [Figure 3I: F_2,184_ = 123.4; P < 0.0001].

### 3.2 Pharmacokinetics of 4F-FUF

#### 3.2.1 Plasma concentration

Urine samples could not be collected due to the antidiuretic effect of 4F-FUF. In this study, we conducted an analysis of the pharmacokinetic profile of 4F-FUF only using samples of the blood (plasma) and various tissues, including the brain, heart, liver, spleen, lung, kidneys, and stomach.

**FIGURE 4:**
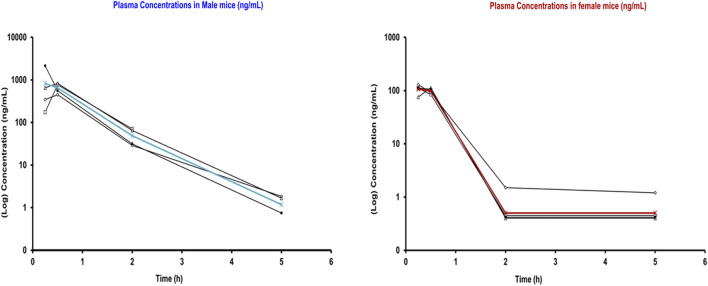
Plasma concentrations (ng/mL) of 4F-FUF in male and female mice. Eight mice (four males and four females) were injected with 4F-FUF (5 mg/kg IP), and plasma samples were used to perform this analysis. Black curves represent the concentrations measured in each sample, while the blue (male) and red (female) curves represent the mean of the concentrations from the four samples.

The plasma concentrations of 4F-FUF remained elevated during the initial hour of analysis for both sexes. In male mice, the concentration of the FA exhibited a tendency to decline in the subsequent hours, reaching very low concentrations at 5 h. In contrast, the plasma concentrations of 4F-FUF in female mice showed a significant drop at 2 h of analysis, and very low concentrations were detected in the following hours (Figure 4).

The pharmacokinetic parameters of 4F-FUF in female and male mice are shown in Table 1. This table demonstrates higher concentrations of 4F-FUF in male mice at 0.25 and 0.50 h than in female mice. The elimination rate constant (K) was slightly higher in males than in females. The apparent elimination half-life was shorter in females (t½ = 0.42 h) than in males (t½ = 0.52 h), which is consistent with the markedly higher apparent clearance observed in female mice.

**TABLE 1:** Pharmacokinetic parameters calculated from various plasma samples of female and male mice treated with 4F-FUF (5 mg/kg IP).

Time (t, h)	Mean concentration in males (ng mL^−1^)	Mean concentration in females (ng mL^−1^)
0.25 h	827	106
0.50 h	650	99
2.00 h	49	0.5
5.00 h	1	0.5
K (h^−1^)	1.380	1.008
t½ (h)	0.52	0.42
Cmax (ng/mL)	1,044.7	115.8
Tmax (h)	0.44	0.31
${\text{AUC}}_{0–\infty }$ (µM.h)	2.54	0.44
CL/F (mL/min/kg)	100.8	782.3
Vd (L/kg)	4.53	28.6

K, elimination rate constant, t½, half-life; Cmax, maximum concentration; Tmax, time to maximum concentration; ${\text{AUC}}_{0–\infty }$ , area under the curve from 0 to ∞ ; Cl, clearance; Vd, volume of distribution.

In male mice, 4F-FUF reached a markedly higher peak plasma concentration than in females, with a mean Cmax of 1,044.7 ng/mL occurring at a Tmax of 0.44 h. Systemic exposure was substantially greater, as reflected by an ${\text{AUC}}_{0–\infty }$ of 2.54 µM·h. The apparent clearance (CL/F) was low (100.8 mL/min/kg), indicating slower elimination of the parent compound. In contrast to females, the apparent volume of distribution (Vd/F) was relatively limited (4.53 L/kg). Overall, these parameters show a different pharmacokinetic profile between female and male mice with a high clearance of 4F-FUF in females than in males, as shown in Figure 4.

#### 3.2.2 Tissue distribution

**FIGURE 5:**
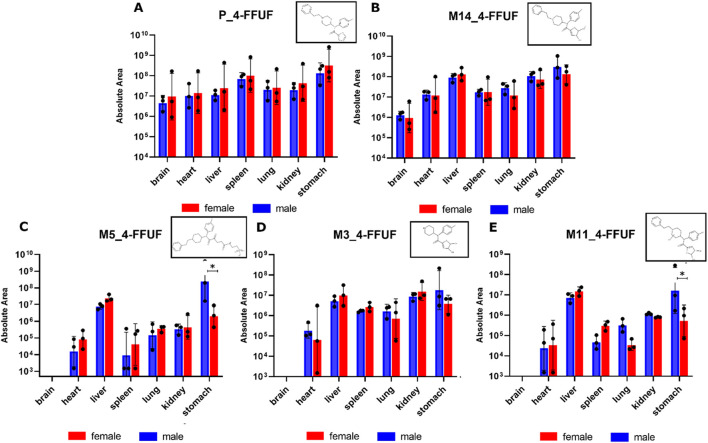
**(A)** Distribution of 4F-FUF (P) in different tissues (brain, heart, liver, spleen, lung, kidney, and Stomach) of female and male mice after treatment with (4F-FUF 5 mg/kg IP). **(B–E)** distribution of the metabolites M14, M5, M3 and M11 in female and male organs. The metabolites were categorized in panels based on their descending order of detection levels. Statistical analysis was performed by unpaired t-test to compare the concentrations in males and females. *p < 0.05 female vs male.

The parent drug (4F-FUF) and four of its major metabolites were detected in tissues from female and male mice (Figure 5). The distribution of the drug and its metabolites appeared to be quite similar in both sexes. The metabolite M14, derived from epoxidation and hydrolysis of 4F-FUF, was most abundant, followed by M5, M3, and M11. Of note, these latter metabolites were not detected in the brain. Statistical analysis revealed significant differences (p < 0.05) in M5 and M11 distribution between sexes, with male mice showing higher levels than females.

#### 3.2.3 Correlation

**FIGURE 6:**
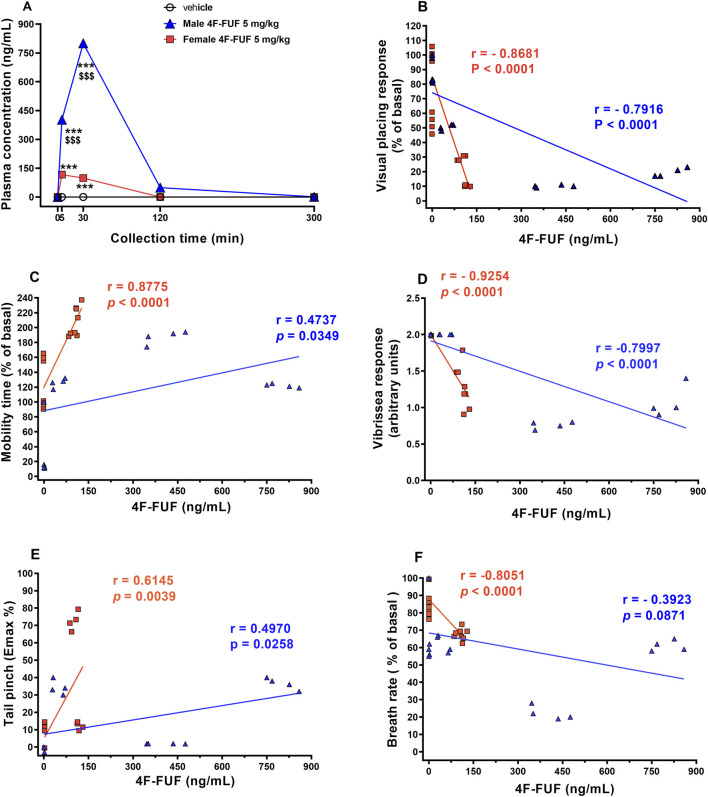
Correlation between plasma concentrations and behavioral and physiological responses of female and male mice treated with 4F-FUF (5 mg/kg IP). Results are expressed as ng/mL and represent the mean ± SEM of four samples for each timepoint. **(A)** Comparison of plasma concentrations in both sexes at 5, 30, 120, and 300 min. Correlation between plasma concentrations in both sexes and visual placing response **(B)**, mobility time **(C)**, vibrissae response **(D)**, tail-pinch **(E)**, and respiratory rate **(F)**. Statistical analysis was performed using two-way ANOVA followed by Bonferroni’s test for multiple comparisons. ***p < 0.001 versus vehicle. $$$p < 0.001 versus sex (female/male). Correlation coefficients were determined by performing Pearson’s correlation calculation.

Plasma concentrations of 4F-FUF in tissue samples of female and male mice are directly correlated with their behavioral and physiological changes (Figure 6). According to Pearson’s calculation, visual placing response (Figure 6B), vibrissae response (Figure 6D), spontaneous locomotion (Figure 6C), and mechanical analgesia (Figure 6E) were significantly correlated with 4F-FUF plasma concentrations in both sexes. Conversely, the respiratory rate (Figure 6F) was only significantly correlated with 4F-FUF plasma concentrations in females but not in males.

## 4 Discussion

### 4.1 Effects of 4F-FUF on sensorimotor, motor, analgesic, and respiratory responses in female and male mice

The results obtained in this study are in accordance with the previous data on the pharmacotoxicology of the FA 4F-FUF (Montesano et al., 2021; Vincenti et al., 2020). Expanding upon the existing knowledge, the data from this current study reveal that 4F-FUF impairs sensorimotor and motor activities with varying intensity in female and male mice. The sensorimotor effects of fentanyl and its analogs (acrylfentanyl, ocfentanil, and furanylfentanyl) were analyzed in our previous study in male mice, and the potential mechanisms underlying these impairments have been discussed, including their translational relevance to humans and their contribution to cases of driving under the influence of drugs (DUID), as reported recently by the United Nations Office on Drugs and Crime (Bilel et al., 2023a). Similarly to fentanyl and its analogs, 4F-FUF may pose a potential risk for driving impairments and might be involved in DUID cases (Rohrig et al., 2021).

4F-FUF impaired the motor responses in female and male mice. In particular, the effect of 4F-FUF was facilitatory at 5 mg/kg in both sexes; however, the effect appeared to be more persistent in males than in female mice. In our previous study on 2-FUF in male mice, we demonstrated that this compound induces stimulatory effects in mice at 1 mg/kg, and the effect was biphasic at the dose of 6 mg/kg [16]. Moreover, FUF induced inhibition of motor activity in male mice at the dose of 6 mg/kg in the accelerod and drag test (Bilel et al., 2022) which was blocked by the pretreatment with naloxone (6 mg/kg), confirming the involvement of the μ-opioid receptors in the motor impairments induced by this compound (Bilel et al., 2022). The differences between the facilitatory effects induced by 4F-FUF and the biphasic or inhibitory effects induced by 2-FUF/FUF could be attributed to the dose tested, the parameter evaluated, and the test employed, such as spontaneous locomotor activity, which differs in terms of task demands and cognitive responses compared with stimulated motor activity (Schönfeld et al., 2017).

The efficacy of 4F-FUF at μ-opioid receptors plays a key role in animals’ motor response. Indeed, a recent study by Santos et al. (2022) found that the hyper-locomotor effect of opioids, particularly fentanyl, depends on their efficacy at the μ-opioid receptors. The mechanisms by which fentanyl and its analogs facilitate locomotion in mice have been fully discussed in our previous studies (Bilel et al., 2022; 2023b; 2025a; Pesavento et al., 2022).

The results from the tail-pinch test revealed sex-specific differences in antinociception. In male mice, the effect of 4F-FUF on mechanical antinociception was moderate (Emax±45%) and persistent until the end of the test (305 min). However, in females, the effect was higher (Emax = 80%) and shorter (90 min). These results further confirm a pharmacokinetic difference between the two sexes, as shown in our results in Figure 4 and Table 1.

It is worth noting that compared to 4F-FUF, FUF induced maximal effects in this test at a lower dose (3 mg/kg) in male mice, confirming a higher efficacy of FUF (Bilel et al., 2022). 4F-FUF is a close derivative of furanylfentanyl (N-phenyl-N-[1-(2-phenylethyl) piperidin-4-yl]furan-2-carboxamide) formed by the addition of a fluorine atom attached to the aniline ring in the para-position (Figure 1). Previous research on fluorinated and non-fluorinated FAs, including 2-fluorofentanyl/fentanyl, butyrylfentanyl/fluoro-isobutyrylfentanyl, and valerylfentanyl/fluoro-valerylfentanyl, indicates that the potency of the fluorinated compounds is similar to or slightly lower (−30%) than that of their non-fluorinated counterparts (Wilde et al., 2019; Varshneya et al., 2023). In addition, studies comparing the potencies of ortho-, meta-, and para-fluorinated fentanyl analogs have shown that the ortho-position exhibits the highest potency, generally followed by the meta- and para-positions (Hassanien et al., 2020; Varshneya et al., 2022). Our results confirm that the substitution of a fluorine atom in the furan ring at the para-position reduces the efficacy of the compounds, resulting in moderate antinociception (Bilel et al., 2022; Varshneya et al., 2023).

Respiratory depression is the most common adverse effect observed with FAs. Our results revealed sex-specific differences in the respiratory rate of mice in response to 4F-FUF (5 mg/kg) treatment. Importantly, a sudden and marked decrease in the respiratory rate (80% reduction) was observed in male mice compared to female mice (30% reduction) at 15 min after injection. These results further confirm sex-specific differences in the pharmacokinetic profile of FAs between female and male mice (Figure 4; Table 1). Previous studies on FUF revealed a decrease in the respiratory rate and hypoventilation in male mice, which was blocked by naloxone, confirming the involvement of μ-opioid receptors in this physiological response (Bilel et al., 2022; Varshneya et al., 2022).

### 4.2 Sex-specific differences in pharmacokinetics of 4F-FUF

The pharmacokinetic analysis of 4F-FUF shows that this opioid was distributed and metabolized differently in male and female mice. Plasma concentrations of 4F-FUF were higher and persisted longer in males, indicating slower clearance than that in females (Figure 4; Table 1). The shorter elimination half-life observed in females is consistent with their substantially higher clearance of 4F-FUF, whereas the longer persistence of the parent compound in males likely contributes to their greater systemic exposure and more pronounced pharmacodynamics effects. The study also examined the distribution of 4F-FUF and its metabolites in various tissues, including the brain, heart, liver, spleen, lung, kidneys, and stomach. The parent compound and its major metabolites were detected in both sexes, with similar distribution patterns. However, notable sex-specific differences in tissue concentrations were observed, particularly in the stomach, which may contribute to the observed differences in pharmacodynamics and toxicological outcomes. These findings indicate that female mice metabolize and eliminate 4F-FUF more rapidly than males. Studies on the pharmacokinetics and pharmacodynamics of fentanyl analogs in female and male mice are scarce (Bilel et al., 2025a). Previous studies indicated that sex-specific differences in fentanyl pharmacokinetics could be related to its sex-specific distribution into adipose tissues (Ohtsuka et al., 2007). In addition, it has been suggested that the differences is attributed to the μ-opioid receptor distribution between both sexes (Zhang et al., 2014). In addition, the effects of circulating ovarian hormones and/or androgens on μ-opioid receptor-mediated physiological responses contribute to the pharmacokinetics differences between both sexes (Sharp et al., 2022). In humans, similar findings were observed with fentanyl and other opioids (Holmquist, 2009). Cytochrome distribution and polymorphism significantly affect the distribution and metabolism of fentanyl in both sexes (Soldin and Mattison, 2009; Gerges and El-Kadi, 2023). Our results underscore the need of evaluating the sex-specific differences in FA pharmacokinetics to better understand their toxicological effects.

### 4.3 Correlation between plasma concentrations of 4F-FUF and behavioral effects

In our study, we explored the correlation between plasma concentrations of 4F-FUF (5 mg/kg IP) and behavioral/physiological changes in female and male mice. Significant correlations were found between plasma levels and behavioral responses, such as visual placing, vibrissae responses, and spontaneous locomotion, in both sexes. Interestingly, the respiratory rate was only significantly correlated with plasma concentrations in females, highlighting potential sex-specific differences in the relationship between drug exposure and physiological effects (Figure 6). It is worth noting that respiratory depression was markedly more pronounced in male mice in the first 10 min after administration, with an approximately 80% reduction in the respiratory rate, compared with a ∼30% reduction in females, despite the absence of a significant correlation between plasma concentrations of 4F-FUF and respiratory effects in males. This apparent dissociation indicates that some factors beyond circulating levels of 4F-FUF may contribute to sex-dependent respiratory depression. In this context, we previously demonstrated that FAs can induce sex-specific differences in respiratory depression, with females exhibiting greater sensitivity at intermediate doses (1 mg/kg IP), indicating that the dose and route of administration critically influence both physiological responses and pharmacokinetic profiles of fentanyl and its analogs (Bilel et al., 2025a; Little and Kosten, 2023; Marchette et al., 2023). One plausible explanation for the present findings is the involvement of pharmacologically active metabolites, whose formation, tissue distribution, or clearance may differ between sexes. Indeed, we have previously shown that 4F-FUF undergoes extensive metabolism *in vivo*, yielding approximately 20 metabolites, with a particularly abundant production of M14_FFUF (Montesano et al., 2021). The reduced plasma persistence of the parent compound in females (Figure 6A), combined with higher clearance (Table 1), may favor faster biotransformation toward active metabolites such as M14, potentially contributing to some physiological effects such as the respiratory depression. In contrast, the dominance of parent compound exposure in males may explain the stronger plasma concentration–effect associations observed for several behavioral endpoints. Further studies are needed to confirm the role of active metabolites in the observed *in vivo* effects.

Given the well-established structure–activity relationships of FAs, even minor structural modifications can substantially influence μ-opioid receptor-mediated respiratory effects (Canfield and Sprague, 2025; Varshneya et al., 2023). Additionally, sex-related hormonal modulation of μ-opioid receptor signaling and central respiratory control circuits may further enhance drug or metabolite availability and efficacy in the brainstem and pulmonary tissues of males (Craft, 2008; Little and Koston, 2023). Together, these mechanisms may contribute to the increased respiratory depression observed in males and underscore the importance of considering both metabolite-driven effects and biological sex when interpreting opioid pharmacotoxicology.

### 4.4 Limitations of the study

Our study presents valuable findings regarding the behavioral and pharmacokinetic effects of 4F-FUF in male and female mice; however, some limitations should be acknowledged. In particular, this study analyzed the effects of a single high dose of 4F-FUF (5 mg/kg), limiting the ability to assess dose-dependent effects and determine the compound’s potency and efficacy across different tests due to resource constraints and efforts to reduce the number of animals used. The pharmacokinetic analyses were performed using a limited sample size per sex and reduced timepoints, which, although consistent with exploratory *in vivo* pharmacotoxicological studies and the 3Rs principle, may limit the estimation of inter-individual variability. Future studies with larger cohorts will be necessary to further refine sex-specific pharmacokinetic parameters.

While the extensive metabolism of 4F-FUF was demonstrated and the potential contribution of active metabolites such as M14_FFUF was discussed, the present study did not directly correlate the behavioral and physiological effects with the excessive metabolites. As such, conclusions regarding active metabolite-driven effects remain unjustified. Dedicated studies focusing on the correlations between metabolites and pharmacological effects are warranted. While behavioral and physiological effects were assessed, the use of opioid receptor antagonists such as naloxone could have provided additional confirmation of 4F-FUF’s mechanism of action. Future studies will incorporate receptor blockade experiments to clarify 4F-FUF’s opioid receptor-mediated effects in both sexes.

## 5 Conclusion

Our study revealed sex-related differences in behavioral and physiological responses, drug metabolism, and tissue distribution between male and female mice, indicating that sex plays a crucial role in the response to FAs. These findings underscore the importance of considering sex-related differences in the pharmacokinetics and pharmacodynamics of NSOs, which could inform future therapeutic strategies and the development of sex-specific guidelines for the use of opioids.

## Data Availability

The original contributions presented in the study are included in the article/Supplementary Material; further inquiries can be directed to the corresponding author.
